# Single‐Cell Integration of BMD GWAS Results Prioritize Candidate Genes Influencing Age‐Related Bone Loss

**DOI:** 10.1002/jbm4.10795

**Published:** 2023-07-07

**Authors:** Madison L. Doolittle, Sundeep Khosla, Dominik Saul

**Affiliations:** ^1^ Division of Endocrinology Mayo Clinic Rochester Minnesota USA; ^2^ Robert and Arlene Kogod Center on Aging Mayo Clinic Rochester Minnesota USA; ^3^ Department for Trauma and Reconstructive Surgery BG Clinic, University of Tuebingen Tuebingen Germany

**Keywords:** AGING, BONE, GWAS, OSTEOPOROSIS, SINGLE‐CELL TRANSCRIPTOMICS

## Abstract

The regulation of bone mineral density (BMD) is highly influenced by genetics and age. Although genome‐wide association studies (GWAS) for BMD have uncovered many genes through their proximity to associated variants (variant nearest‐neighbor [VNN] genes), the cell‐specific mechanisms of each VNN gene remain unclear. This is primarily due to the inability to prioritize these genes by cell type and age‐related expression. Using age‐related transcriptomics, we found that the expression of many VNN genes was upregulated in the bone and marrow from aged mice. Candidate genes from GWAS were investigated using single‐cell RNA‐sequencing (scRNA‐seq) datasets to enrich for cell‐specific expression signatures. VNN candidate genes are highly enriched in osteo‐lineage cells, osteocytes, hypertrophic chondrocytes, and Lepr+ mesenchymal stem cells. These data were used to generate a “blueprint” for Cre‐loxp mouse line selection for functional validation of candidate genes and further investigation of their role in BMD maintenance throughout aging. In VNN‐gene‐enriched cells, *Sparc*, encoding the extracellular matrix (ECM) protein osteonectin, was robustly expressed. This, along with expression of numerous other ECM genes, indicates that many VNN genes likely have roles in ECM deposition by osteoblasts. Overall, we provide data supporting streamlined translation of GWAS candidate genes to potential novel therapeutic targets for the treatment of osteoporosis. © 2023 The Authors. *JBMR Plus* published by Wiley Periodicals LLC on behalf of American Society for Bone and Mineral Research.

## Graphical abstract

The goal of this manuscript is to identify the cellular source of BMD GWAS candidate genes and provide a streamlined approach to translate these findings to new therapeutic targets and illustrate suitable Cre‐loxp mouse models. As such, the insights from this work should greatly facilitate the ability to identify cellular targets of novel anti‐osteoporotic treatments.

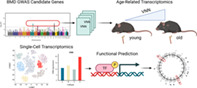

## Introduction

Osteoporosis is a chronic disorder of low bone mass and increased fracture risk that is strongly influenced by genetics, with a high prevalence in aged individuals. Up to one out of three individuals over the age of 50 will suffer a fragility fracture,^(^ ^1^ ^)^ which increases overall mortality risk due to existing comorbidities.^(^ ^2^ ^)^ Bone mineral density (BMD), the diagnostic metric for osteoporosis, is a complex trait largely influenced by genetic variance, with heritability estimates for BMD observed as high as 89%.^(^ ^3^ ^)^ Indeed, the biggest risk factor of developing osteoporosis is family history.^(^ ^4^, ^5^ ^)^ It has been shown that genetic effects are exerted on peak bone mass^(^ ^6^ ^)^ as well as rates of age‐related bone loss.^(^ ^7^ ^)^ Therefore, genetic studies focusing on bone traits have been a major focus in the field to identify genes that may regulate bone strength in aged individuals.

Genome‐wide association studies (GWAS) have uncovered vast amounts of information to understand the genetic regulation of BMD. Over the last two decades, GWAS have identified over 1000 associated single nucleotide polymorphisms (SNPs) underlying over 500 genomic loci associated with BMD, explaining up to 20% of the variance in this phenotype.^(^ ^8^, ^9^, ^10^ ^)^ These loci seek to identify candidate genes that may regulate bone metabolism, typically reported as genes closest in proximity to the associated variant (variant nearest‐neighbor [VNN] genes). Initial studies characterizing these genes found roles in bone maintenance using mouse knockout models.^(^ ^10^, ^11^ ^)^ Additionally, recent work by Kaya et al. prioritized GWAS genes associated with aging and fracture risk through integration with bulk RNA sequencing (RNA‐seq) across murine aging.^(^ ^12^ ^)^ Although important, global knockout models and bulk RNA‐seq fail to resolve cell‐specific gene actions, and thus our understanding of how each gene mechanistically regulates bone density remains incomplete. Specifically, it is unclear whether these genes lead to primary (bone‐intrinsic) or secondary (bone‐extrinsic or systemic) alterations in bone metabolism. Even if the gene is linked to bone formation (executed by the osteoblast), the osteogenic lineage is highly heterogeneous,^(^ ^13^, ^14^, ^15^, ^16^ ^)^ leading to complexity even within broad cell categories. Cell‐specific gene expression analyses for each candidate gene would greatly augment experimental design for functional follow‐up studies using tissue‐ and cell‐specific knockouts, which may generate novel therapeutic targets for the treatment of osteoporosis. However, until this is accomplished, the further characterization of GWAS‐associated candidate genes will remain limited.

In this study, we addressed this problem by investigating the cell‐ and age‐specific gene expression profiles of GWAS VNN genes in established bone cell types. We leveraged both bulk and single‐cell RNA‐seq (scRNA‐seq) datasets to pinpoint cell‐specific gene expression of current candidate genes in addition to their age‐related expression profile. We aim to have our data serve as a “blueprint” for future mechanistically driven functional studies of GWAS‐associated genes, with the goal of streamlining the genetic discovery pipeline from genomic association to therapeutic target.

## Materials and Methods

### Curation of GWAS VNN candidate genes

Candidate genes were derived from the results of a previously published GWAS dataset.^(^ ^10^ ^)^ Briefly, this study identified 1103 independent SNPs within 515 genomic loci associated with calcaneal estimated bone mineral density (eBMD) using the UK Biobank cohort of 426,824 participants. Candidate genes were selected as the gene closest to the associated variant with the smallest *p* value of all conditionally independent variants within the same locus, generating 514 human candidate genes. Mouse orthologs were identified (SYNGO,^(^ ^17^ ^)^ bioDBnet^(^ ^18^ ^)^), while removing noncoding and microRNAs, resulting in 436 candidate genes for downstream analyses in murine datasets (Supplementary Table S1).

### Analysis of mRNA sequencing data

The enrichment of VNN genes was tested in two publicly available bulk mRNA‐Seq datasets (bone and bone marrow: Tabula Muris Consortium GSE132040,^(^ ^19^ ^)^ bone: *n* = 12 [months 6–9] young versus *n* = 14 old [months 21–27]; marrow: *n* = 11 young [months 6–9], *n* = 13 old [months 21–27]).

Raw counts were converted into a matrix before DESeq2 (1.34.0) was used. Determination of differentially expressed genes (DEGs) was performed using DESeq2 (lfcThreshold = 0, alpha = 0.1, minimum count = 0.5). An example of an RNA‐seq analysis vignette is provided as R notebook in our previous study.^(^ ^20^ ^)^ Gene Set Enrichment Analysis (GSEA, version 4.2.2, Broad Institute, Inc., Massachusetts Institute of Technology, Cambridge, MA, USA, and Regents of the University of California, Berkeley, CA, USA) was performed with default settings (1000 permutations for gene sets, Signal2Noise metric for ranking genes).

### scRNA‐seq analysis

Enrichment of VNN genes in distinct cell populations was determined by analyzing a publicly available single‐cell sequencing dataset from Baryawno et al. (GSE128423^(^ ^15^ ^)^), as demonstrated in Saul et al.^(^ ^21^ ^)^

Cells with at least 500 unique molecular identifiers (UMIs), log10 genes per UMI >0.8 and >250 genes per cell, and a mitochondrial ratio of less than 20% were extracted, normalized, and integrated using the Seurat package (version 4.0.6) in R 4.0.3.

The cell annotation was provided by the authors. Detailed information on sample characteristics, conditions, and cell numbers per cluster from the bone and marrow scRNA‐seq dataset is summarized in Supplementary Table S2.

The normalization, scaling, and clustering followed the recommendations of the Seurat package.^(^ ^22^ ^)^ In particular, normalization and scaling were performed with NormalizeData and ScaleData, followed by an SCTransformation. Comparisons between TOP25 and BOTTOM75 samples were performed using the “FindMarkers” function (Seurat package) and the MAST package (1.16.0,^(^ ^23^ ^)^ logfc.threshold = 0, test.use=”MAST”, only.pos = FALSE, min.pct = 0.0). For pairwise comparisons, a nonparametric Wilcoxon signed‐rank test was applied (ggpubr 0.4.0). The top genes per cluster are demonstrated in Table 1. GSEA was conducted using clusterProfiler (3.18.1). Heatmaps were designed using the DEP package (1.1.5). Column bars were created using the scater package (1.18.6). Bubble plots were designed with the ggplot2 (3.3.5) package, while regulatory elements were identified following the SCENIC package recommendations (1.2.4,^(^ ^24^ ^)^).

**Table 1:** Cell‐Type Categorization of GWAS VNN Genes Based on scRNA‐seq Cluster Enrichment, Annotated with Cre‐Loxp Mouse Models for Cell‐Specific Functional Validation of Each Gene

Cluster	Gene	Log2FC cluster expression	In vivo Cre‐Loxp system
Pericytes	Bcas3	1.519	Lepr‐Cre,^(^ ^48^ ^)^ Tagln‐Cre,^(^ ^49^ ^)^ Nestin‐Cre^(^ ^50^ ^)^/‐CreERT2^(^ ^51^ ^)^
	Ppp1cb	1.238	
	Zfhx3	1.138	
	Synpo2	1.072	
	Nab1	0.803	
	Irs2	0.709	
	Inpp5a	0.635	
	Abr	0.580	
	Chd4	0.345	
	Trim2	0.329	
	Cnot4	0.295	
Endothelial cells	Kdr	2.138	Tie2‐Cre^(^ ^52^ ^)^/‐CreERT2,^(^ ^53^ ^)^ Cdh5‐Cre^(^ ^54^ ^)^/‐CreERT2^(^ ^55^ ^)^
	Ehd4	1.341	
	Tcf4	1.264	
	Sptbn1	1.216	
	Plpp1	1.144	
	Calcrl	0.902	
	Lrrc8c	0.891	
	Tgfbr2	0.882	
	Jup	0.872	
	Cmip	0.851	
	Palmd	0.830	
	Ctnnb1	0.764	
	Dab2	0.744	
	Tanc1	0.645	
	Nfe2l1	0.408	
	Tulp4	0.396	
	Mkln1	0.313	
Fibroblast	Col1a2	0.985	Fsp1(S100a4)‐Cre,^(^ ^56^ ^)^ Acta2‐Cre^(^ ^57^ ^)^
	Axl	0.958	
	Aqp1	0.948	
	Ntn1	0.676	
	Sema3e	0.579	
	Prrx1	0.556	
	Ahnak	0.461	
	Emp1	0.457	
	Klf4	0.455	
	Tmem119	0.404	
	Thbs3	0.397	
	Rhoj	0.371	
	Itgb5	0.320	
Lepr MSC	H2‐K1	1.851	Lepr‐Cre,^(^ ^48^ ^)^ Cxcl12‐CreERT2^(^ ^58^ ^)^
	H2‐D1	1.591	
	Ghr	1.291	
	H2‐Q7	1.216	
	Plpp3	1.215	
	Ptprd	1.205	
	H2‐Q10	1.181	
	Ebf1	1.154	
	Gja1	1.039	
	Tgfbr3	1.009	
	Mpdz	0.915	
	Plxdc2	0.901	
	Bicc1	0.901	
	Klf6	0.893	
	Fosb	0.814	
	Bmp4	0.783	
	Rbms3	0.762	
	Dlc1	0.709	
	Gpc6	0.701	
	H2‐Q4	0.594	
	Klf9	0.461	
	Tcf7l2	0.446	
	Med13l	0.441	
	Rbpj	0.431	
	Emp2	0.407	
	Nfia	0.372	
	Stard3nl	0.322	
MSC	Mgmt	1.505	Prrx1‐Cre,^(^ ^59^ ^)^ Cxcl12‐CreERT2,^(^ ^58^ ^)^ Pdgfra‐CreERT2,^(^ ^60^ ^)^ Pdgfrb‐Cre^(^ ^61^ ^)^
	Cotl1	1.403	
	Dnmt3a	1.188	
	Fli1	1.066	
	Myh9	0.951	
	Capzb	0.746	
	Fbxl2	0.559	
	Ptprj	0.444	
	Meis1	0.443	
	Prelid1	0.438	
	Ube2l3	0.431	
	Litaf	0.397	
	Ank3	0.365	
	Cyfip1	0.348	
	Hspa4	0.294	
OLC 1	Lrp4	1.728	Col1a1‐Cre,^(^ ^62^ ^)^ Osx‐Cre^(^ ^63^ ^)^/‐CreERT2,^(^ ^64^ ^)^ Runx2‐Cre, Ocn‐Cre^(^ ^65^ ^)^
	H2‐Q6	0.939	
	Fat1	0.908	
	Esr1	0.613	
	Wls	0.488	
	Adam12	0.461	
	Sema6d	0.454	
	Foxn3	0.365	
	Epb41l2	0.360	
	Irf2bp2	0.292	
	Bmp5	0.282	
OLC 2	Smoc1	0.774	
	Frzb	0.557	
Osteocyte	Mettl7a1	0.346	Dmp1‐Cre^(^ ^66^ ^)^/‐CreERT2,^(^ ^67^ ^)^ Sost‐Cre^(^ ^68^ ^)^/‐CreERT2^(^ ^69^ ^)^
	Zbtb38	0.328	
Mineralizing osteocyte	Tnfrsf11b	1.937	
	Mgp	1.486	
	D630045J12Rik	0.947	
	Ngef	0.665	
	Trps1	0.558	
	Tmed10	0.399	
	Tmem43	0.386	
	Mbnl1	0.363	
	Msmo1	0.314	
	Cd109	0.296	
	Pdgfc	0.282	
	Meox2	0.280	
	Nfix	0.272	
	Ltbp3	0.267	
	Etfa	0.259	
	Iqgap1	0.256	
Chondro‐prol/rest	Bcl11a	0.742	Sox9‐Cre,^(^ ^70^ ^)^ Col2a1‐Cre^(^ ^71^ ^)^/‐CreERT2^(^ ^72^ ^)^
	Rangap1	0.522	
	Baz1a	0.425	
	Psmb3	0.391	
	Fam111a	0.381	
	Zfp800	0.271	
	Ubap2	0.258	
	Naa38	0.255	
Chondro‐progen	Smoc1	0.774	
	Frzb	0.557	
Chondrocyte	E2f1	1.102	
	Stk3	0.746	
	Trim27	0.645	
	Hdac4	0.479	
	Cdh6	0.388	
	Zfp113	0.337	
	Mepe	0.335	
	Irx5	0.302	
	Hecw2	0.270	
	Eya1	0.265	
Chondro‐hyper	Col11a1	2.607	Acan‐CreERT2,^(^ ^73^ ^)^ Col10a1‐Cre^(^ ^74^, ^75^ ^)^
	Papss2	1.992	
	Cox4i2	0.832	
	Ptch1	0.830	
	Rab28	0.672	
	Grb10	0.605	
	Sox5	0.544	
	Cdk6	0.532	
	Rspo3	0.487	
	Sp7	0.416	
	Mrps28	0.349	
	Tmem263	0.345	
	Hs3st3b1	0.345	
	Kcnma1	0.335	
	Dlx5	0.325	
	Sema3d	0.312	
	Sobp	0.296	
	Itpr2	0.294	
	Supt3	0.268	
	Fgfrl1	0.267	
Lymphocyte	Arhgap15	0.790	Lck‐Cre,^(^ ^76^ ^)^ Thy1‐Cre,^(^ ^77^ ^)^ Cd19‐Cre^(^ ^78^ ^)^
	Dock8	0.766	
	Rbm5	0.719	
	Stk10	0.563	
	Nt5c2	0.505	
	Smad7	0.403	
	Ctps	0.315	
	Arid1a	0.314	
	Smarcad1	0.282	
	Rere	0.281	
	Foxk2	0.263	
	Atxn7l1	0.261	

### Genome visualization

The murine genome mm10 (Grcm38) was depicted with biovizBase (version 1.44) and GenomicRanges (version 1.48), as well as ggbio (version 1.20.1). The genomic location of marker genes was taken from the Genome Reference Consortium (National Institutes of Health [NIH], date: January 9, 2012). The skeletal phenotype was assessed by the references from Table 2.

**Table 2:** Summary of Current Skeletal Phenotype Data for GWAS VNN and Coexpressed Genes

Gene symbol	Main cell type	Skeletal role	Reference
*Mgp*	Mineralizing osteocyte	+	(25)
*Col1a2*	Fibroblast, Chondro‐hyper	+	(26)
*Col11a1*	Chondro‐hyper	+	(27)
*Ebf1*	Lepr MSC	+	(28)
*Klf4*	Fibroblast	−	(29)
*Aqp1*	Fibroblast	+/−	(30, 31)
*Gja1*	Lepr MSC	+	(32)
*Tcf4*	Endothelial cells	+	(33)
*Tnfrsf11b*	Mineralizing osteocyte	+	(34)
*Ctnnb1*	Endothelial cells	+	(35)
*Nfix*	Mineralizing osteocyte	−	(36)
*Malat1*	Lepr MSC	+	(37)
*Sparc*	Chondro‐hyper	+	(38)
*Dcn*	Mineralizing osteocyte	+	(39)
*Cst3*	Chondro‐prehyper	+	(40)
*Fth1*	Fibroblast	−	(41)
*Spp1*	OLC1	−	(42)
*Cytl1*	Chondro‐progen	−	(43)
*Prg4*	Chondro‐progen	+	(44)
*Fos*	Osteocyte	−	(45)

Abbreviation: MSC = mesenchymal stem cell.

### Statistics and graphs

Statistical analyses were performed using a D'Agostino–Pearson test for normality. If the D'Agostino‐Pearson test was passed, an unpaired *t*‐test was performed. Otherwise, a Mann–Whitney test was performed (*p ≤ 0.05, **p ≤ 0.01, ***p ≤ 0.001). Correlation analyses were performed with Spearman's correlation.

Graphs were designed using GraphPad Prism 9.2.0 (GraphPad Software, Inc., San Diego, CA, USA), BioRender.com, and R (4.0.3).

## Results

### Age‐dependent assessment of VNN‐associated genes

**Fig. 1:**
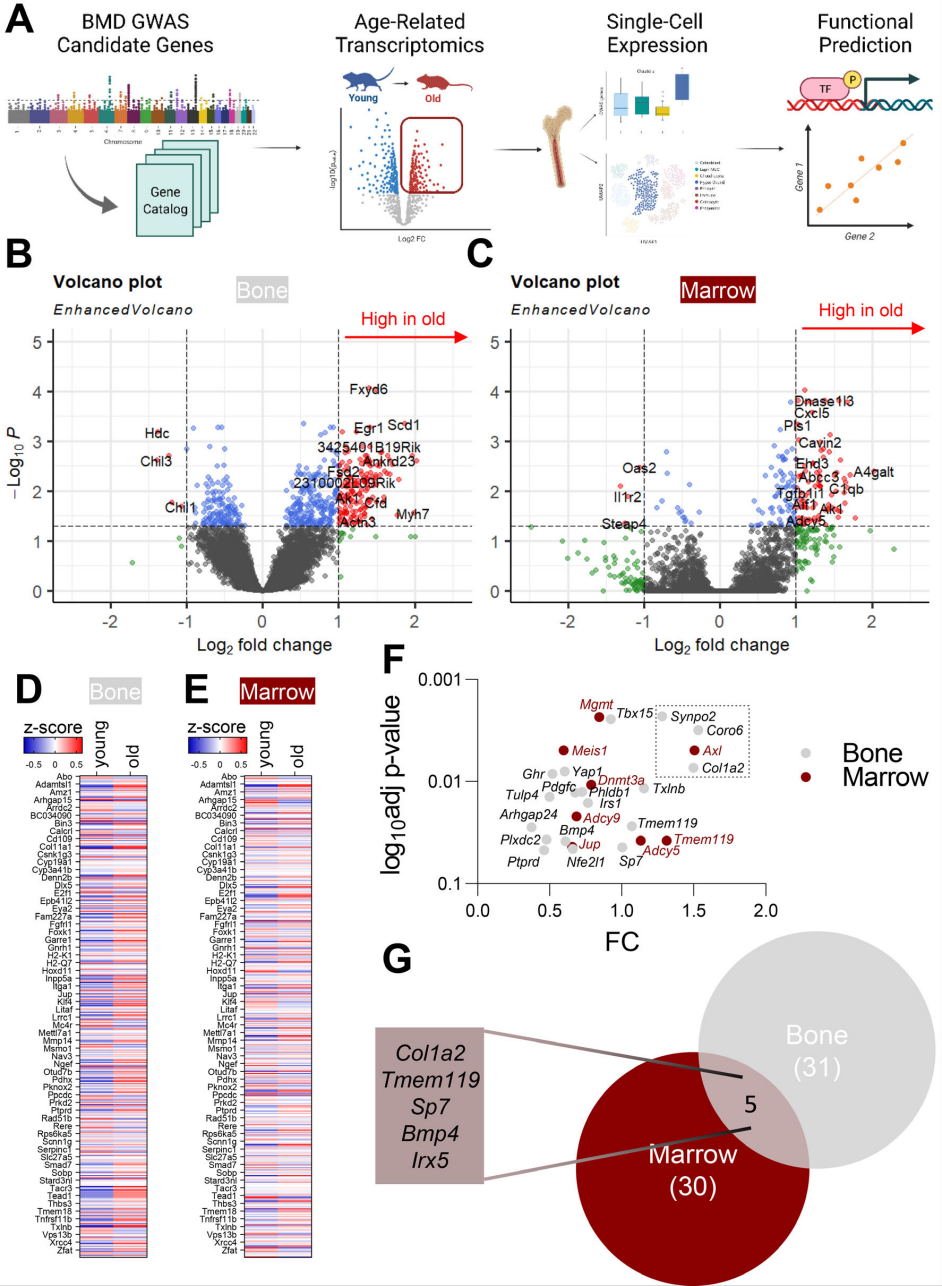
GWAS VNN candidate gene expression increases in bone and marrow in old mice. (*A*) Schematic workflow demonstrating experimental approach. (*B, C*) In both bone and bone marrow, a vast number of genes are upregulated in aging (GSE132040 ^(^ ^19^, ^46^ ^)^, bone: *n* = 12 [months 6–9] young versus *n* = 14 old [months 21–27]; marrow: *n* = 11 young [months 6–9], *n* = 13 old [months 21–27]). (*D*) All GWAS‐associated genes show an upregulation in young versus old mouse bone (two‐way ANOVA *p* < 0.0001) and (*E*) mouse bone marrow (two‐way ANOVA *p* = 0.0085). (*F*) The top significantly increased genes from the VNN genes (*p*_adj_ < 0.05) and all genes with a >0.5 FC fold‐change (*G*) in bone and marrow as their overlap are displayed as a VENN diagram.

Our list of GWAS VNN candidate genes was curated from a recent study,^(^ ^10^ ^)^ as detailed in the Methods. To investigate which genes impact the aging bone microenvironment, we screened VNN candidate genes for age‐dependent differential expression (Fig. 1*A*). We utilized the extensive bone and marrow RNA‐seq datasets from the *Tabula Muris Senis*, which provides a transcriptomic atlas of aging mouse tissues.^(^ ^10^, ^46^ ^)^ In both the bone (Fig. 1*B*) and bone marrow (Fig. 1*C*), the vast majority of genes were enriched in aged (21‐ to 27‐month‐old) compared to young (6‐ to 9‐month‐old) animals. We next assessed all 436 of our VNN candidate genes with regard to young versus old bone (Fig. 1*D*) and bone marrow (Fig. 1*E*). In both compartments, VNN genes showed a significant enrichment in old mice (two‐way ANOVA: *p* < 0.0001 and *p* = 0.0085, respectively). When used as a gene set, a significant enrichment in the old compared to the young cohort was detected for the VNN gene list (Fig. S1 *A,B*. Bone: NES 1.80, *p* value 0.0; marrow: NES 1.47, *p* value: 0.0). The VNN list also shows a significant upregulation with age in bone (*p* value 0.004), while a significant difference is missed in marrow (*p* = 0.289, Fig. S1 *C,D*). When just the significant genes were sorted according to their fold‐change (Fig. 1*F*), *Synpo2*, *Coro6*, and *Col1a2* within the bone and *Axl* within the marrow environment appeared as the top hits. When overlapping the upregulated genes from each compartment with at least a fold‐change of 0.5, just five genes were among the top hits (*Col1a2*, *Tmem119*, *Sp7*, *Bmp4*, *Irx5*) (Fig. 1*G*). In summary, GWAS VNN candidate genes exhibited upregulated expression with age in both bulk sequencing compartments (bone: 18 out of 434 with *p*_adj_ < 0.05 and marrow: 8 out of 434 with *p*_adj_ < 0.05), generating an additional brief list of top hits in both compartments, with little overlap.

### Single‐cell sequencing reveals cellular origin of GWAS VNN genes

**Fig. 2:**
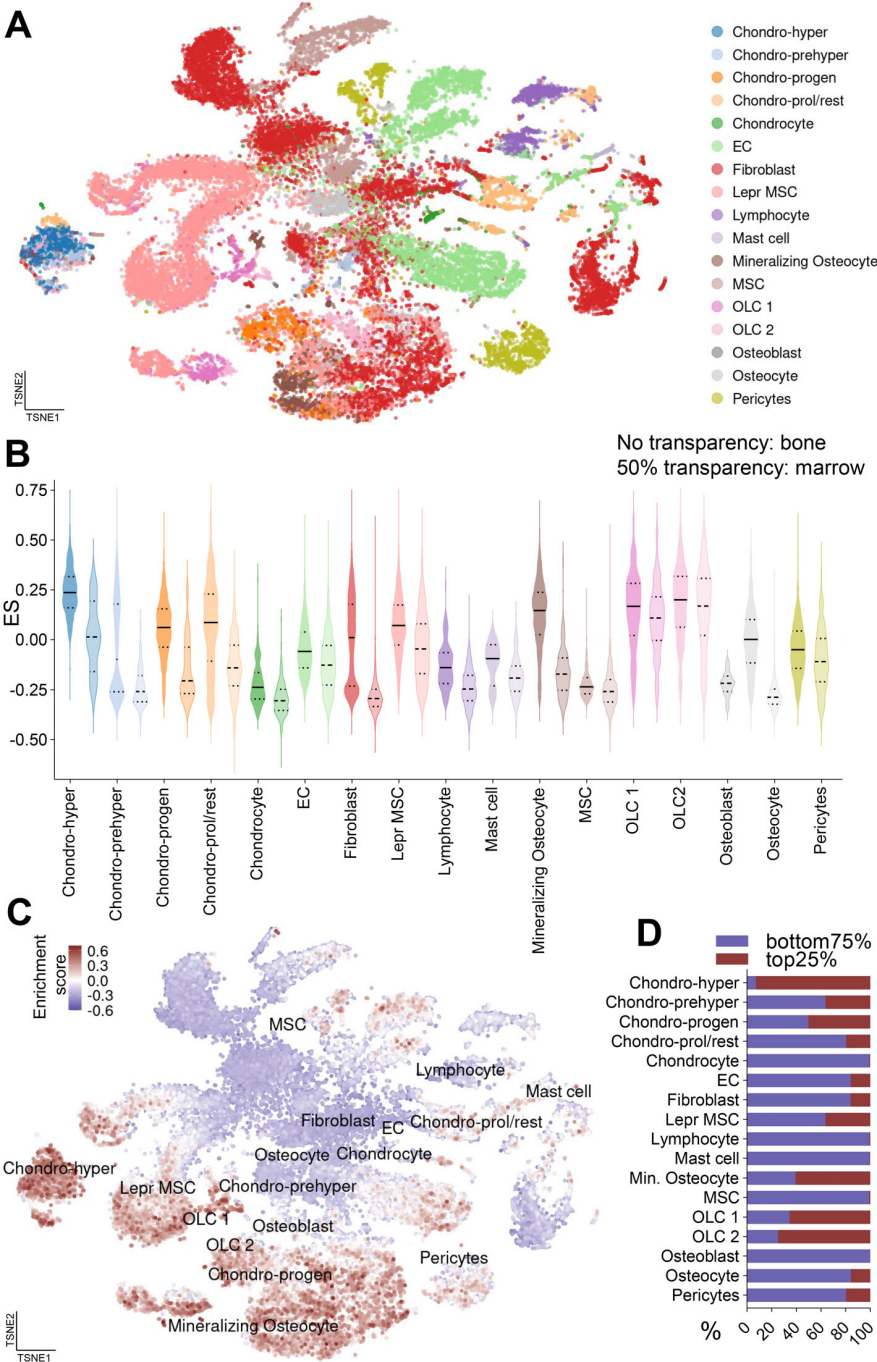
GWAS VNN genes are enriched in committed mesenchymal cell types. (*A*) We used the scRNA‐seq dataset from Baryawno et al. consisting of 35,368 cells in 17 distinct cellular clusters, depicted as a *t*‐distributed stochastic neighbor embedding (tSNE) (^(^ ^15^ ^)^, GSE128423). (*B*) Within these 17 clusters, the VNN genes were enriched in both bone and marrow (50% transparency: marrow). The overall highest enrichment occurs within hypertrophic chondrocytes, mineralizing osteocytes, and osteo‐lineage cell types 1 and 2 (OLC1, OLC2). (*C*) The enrichment score is plotted on the tSNE, demonstrating the higher enrichment of certain mesenchymal populations, which (*D*) account for the majority of the top 25% of VNN‐gene‐enriched cells. Summarizing, the mesenchymal cells were endued with overall higher enrichment, while hypertrophic chondrocytes and OLC2 contained the highest percentage of VNN‐gene expression.

Next, we aimed to verify the expression profile of each candidate gene at single‐cell resolution. For this purpose, we utilized a recently published scRNA‐seq dataset containing bone and marrow cells isolated from eight C57Bl/6 mice (age: 8 to 10 weeks) (Fig. 2*A*).^(^ ^15^ ^)^ We present the clustering and marker genes elsewhere.^(^ ^21^ ^)^ Cells with high expression of our 436 GWAS VNN candidate genes were enriched within the 35,368 total cells in this dataset. Interestingly, hypertrophic chondrocytes, mineralizing osteocytes, and osteo‐lineage cells (OLC1 and OLC2) showed the highest overall enrichment scores (Fig. 2*B*). We noted a tendency of mesenchymal cell types to enrich higher in the VNN genes, particularly in committed cell populations (e.g., mineralizing osteocyte, chondro‐hyper, OLC1/2) (Fig. 2*C*). The selection of a “top25%” cluster, which contains the top 25% of cells with the highest VNN enrichment score, demonstrated a high percentage of cells within the hypertrophic chondrocyte and OLC2 clusters (Fig. 2*D*). These data demonstrated that GWAS VNN genes tended to be expressed in committed osteo‐ and chondrogenic cell types rather than stem or immune cells, with the exception of Lepr mesenchymal stem cells (MSCs). By just enriching the significantly upregulated genes with aging (Fig. S3 *A*), or even the top genes (Fig. S3 *B,C*), the enrichment focuses on the same populations as suggested in Fig. 2*C*. This suggests that, while the level of gene expression differs throughout aging, the cell types expressing VNN genes remain largely consistent.

Using these data, we assigned cell‐specific *in vivo* Cre‐recombinase mouse models for the functional downstream analysis of candidate gene mechanisms (Table 1, extended with top expressed genes as Supplementary Table S1). We should emphasize that, although each Cre‐ model can specifically target the described cell type, each Cre may recombine in unintended cell types. For example, the Pdgfrb‐Cre (fibroblast) has been shown to also recombine in adipocyte progenitors, pericytes, and MSCs.^(^ ^47^ ^)^ Therefore, proper lineage tracing, controls, and follow‐up experiments should be applied before conclusions are made.

### Cellular composition of key regulatory genes predicted by VNN genes

**Fig. 3:**
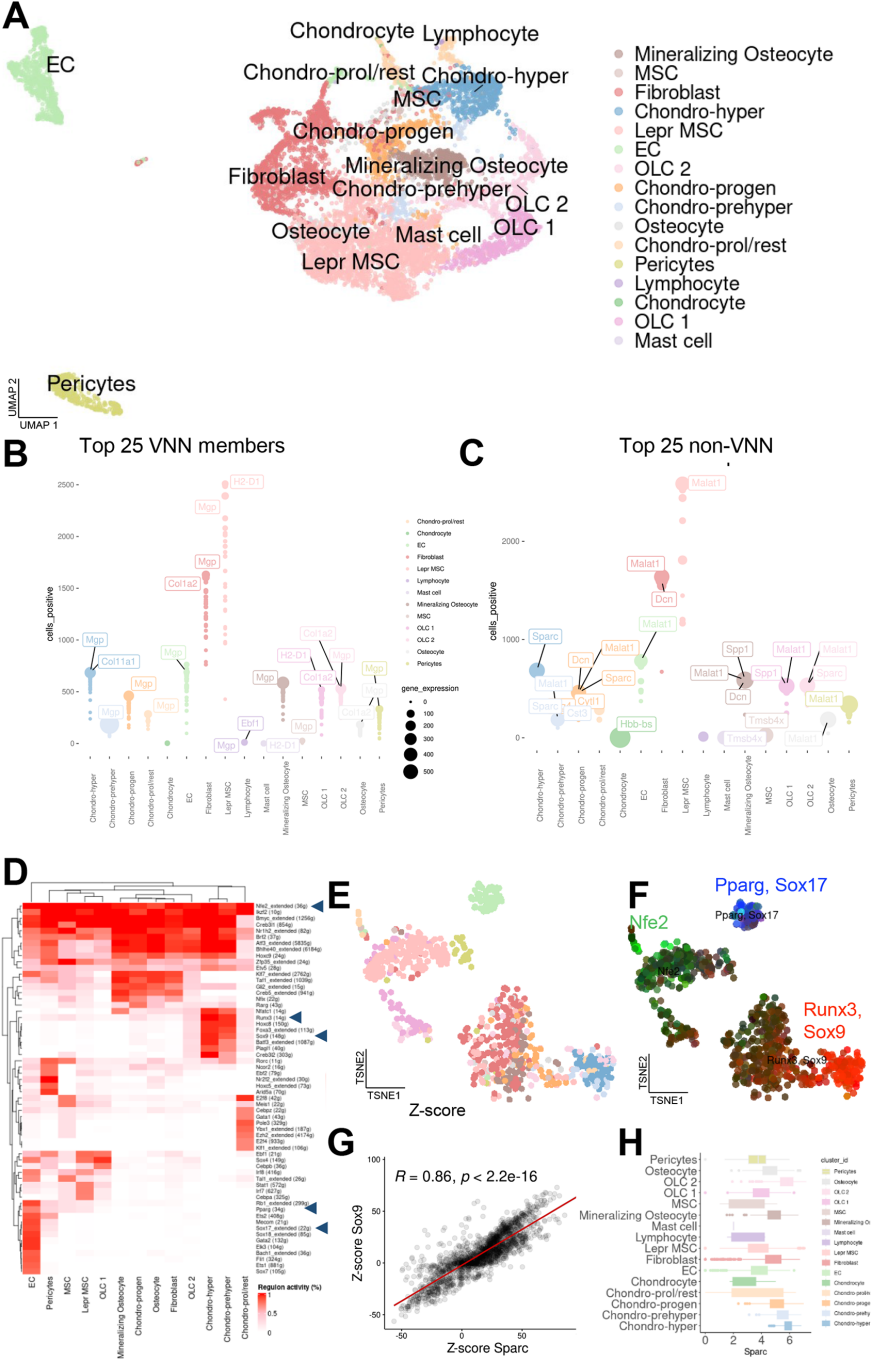
Characterization of top 25 GWAS VNN‐gene‐enriched cells. (*A*) Top 25% VNN‐gene‐enriched cells (*n* = 8842) and cell clusters plotted in UMAP coordinates. (*B, C*) Gene expression (*y*‐axis and dot size) and number of positive cells (*x*‐axis) in each cluster (color code same as in *A*) for the top 25% VNN (*B*) and non‐VNN coexpressed (*C*) genes. (*D*) The regulatory elements of each cluster are demonstrated with the regulon activity per cluster and regulator colored in red. Arrowheads point to the key regulators used for further analysis. (*E*) Key regulators were used to calculate a tSNE representation of all cell types from A. (*F*) The key regulators *Pparg* and *Sox17* are shown in blue, *Nfe2* in green, and *Runx3* as *Sox9* in red, visualized on the regulator‐based tSNE. (*G*) The regulating element *Sox9* correlates with *Sparc* expression within all top 25% GWAS VNN‐gene‐expressing cells (*R* = 0.86, *p* < 0.0001). (*H*) *Sparc* expression is highest in hypertrophic chondrocytes and osteolineage cells (OLC 2).

The top 25% VNN‐enriched cells were separately analyzed, and within these, MSCs, hypertrophic chondrocytes, and OLC 1 and OLC2 cells represent the largest proportions (Fig. 3 *A*, Supplementary Fig. S2). To verify which of the 436 GWAS VNN genes were of greatest importance, we compared the total gene expression per cluster and the total cells within each cluster that expressed these genes (Fig. 3 *B*, Fig. S5 *A*). The gene *Mgp* was substantially upregulated in many cell types: Hypertrophic and prehypertrophic chondrocytes, mineralizing osteocytes, and chondrocytic progenitors exhibited a high expression of *Mgp*, while the expression was lower in fibroblasts and Lepr MSCs, although a high percentage of cells within these clusters expressed *Mgp*.

To investigate additional genes that may have roles in cells influenced by VNN genes, we identified strongly coexpressed genes within the top 25% VNN gene‐enriched cells, termed “non‐VNN co‐expressed” genes. Pericytes, fibroblasts, and Lepr MSCs expressed high levels of *Malat1*, a long noncoding RNA with established roles in regulating angiogenesis^(^ ^79^ ^)^ and osteogenesis,^(^ ^80^, ^81^ ^)^ with its upregulation shown to alleviate ovariectomy‐induced bone loss in mice.^(^ ^37^ ^)^ Within the hypertrophic chondrocytes and mineralizing osteocytes, *Sparc* and *Dcn*, respectively, showed the highest expression (Fig. 3 *C*, Fig. S5 *B*). *Sparc*, encoding the protein osteonectin, is a secreted ECM protein known to be expressed in osteoblasts and chondrocytes^(^ ^82^ ^)^ that is critical for osteogenesis; *Sparc* knockout mice exhibit an age‐dependent reduction in bone quantity,^(^ ^38^ ^)^ intervertebral disc degeneration,^(^ ^83^ ^)^ cataracts,^(^ ^84^ ^)^ and adiposity.^(^ ^85^ ^)^ *Dcn* encodes the ECM protein decorin, which is found in all major type I and II collagen matrices, particularly in the skeleton.^(^ ^39^ ^)^ Therefore, cells positive for these marker genes may be most relevant in their intrinsic expression of VNN genes, suggesting that GWAS candidate gene functions are strongly linked to regulating ECM production.

A deeper analysis of these VNN‐gene‐enriched cell types was performed by regulatory network interference and clustering using the SCENIC package.^(^ ^86^ ^)^ We found the key regulators for each cluster and reordered the cell types based on their regulatory units. While *Nfe2* shows the largest area under the curve (AUC) coverage of MSCs, reaching within the mineralizing osteocyte cluster, *Pparg* and *Sox17* mostly cover endothelial cells (Fig. 3*D*–*F*). All of the regulons are separately shown in Supplementary Fig. S4. Although Pparg is an adipogenic regulator, it has also been observed to be expressed in endothelial cells in other scRNA‐seq datasets of the bone microenvironment.^(^ ^87^ ^)^ Interestingly, *Runx3* and *Sox9* mostly influence hypertrophic and prehypertrophic chondrocytes (Fig. 3*E,F*). To further validate the relevance of *Sox9* and *Sparc* for the osteo‐lineage cells and chondrocyte populations, we analyzed the coexpression of the regulating element (*Sox9*) with the osteonectin coding gene (*Sparc*), which was highly significant (*R* = 0.86, *p* < 0.0001, Fig. 3*G*). The overall *Sparc* expression was indeed highest in these two clusters, but *Sparc* was expressed at a certain level ubiquitously, demonstrating its widespread role in osteogenesis (Fig. 3*H*).

**Fig. 4:**
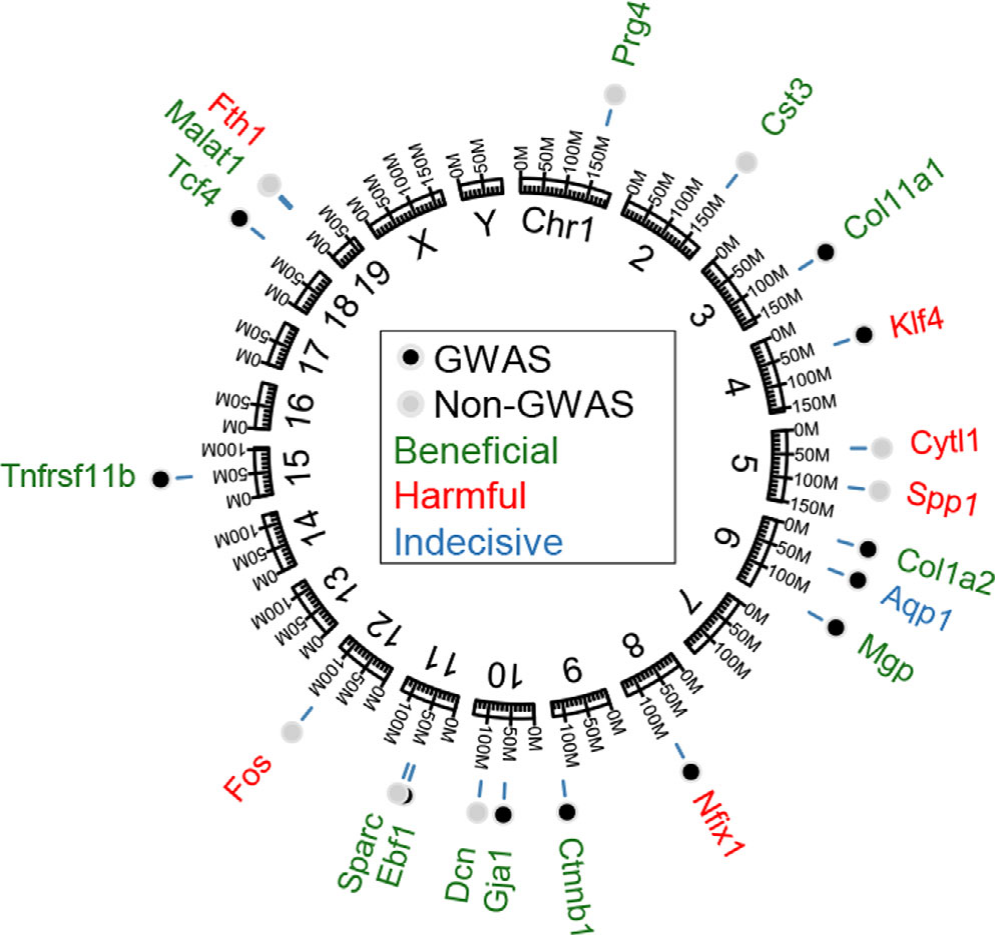
GWAS VNN and non‐VNN coexpressed genes in the murine genome and their impact on skeletal homeostasis. Genome coordinates from the highest expressing genes, demonstrating VNN (black dot) and non‐VNN coexpressed (gray dot) genes that were found within the literature to be beneficial (green font), harmful (red font), or reported to be both/indecisive (blue font) on the murine skeleton (Table 2).

The top 50 genes in the VNN‐ and non‐VNN‐gene groups were further characterized based on their effect on the skeletal phenotype according to the literature. For 20 of these genes, previous mechanistic studies confirmed roles for each gene in the regulation of bone mass as beneficial, harmful, or indecisive (contradicting results) (Fig. 4, Table 2).

In summary, by more extensively characterizing GWAS VNN‐gene enriched cell populations, we were able to identify key regulatory units that may putatively direct osteogenic gene expression. The osteo‐lineage population showed a *Sox9*‐enhanced upregulation of *Sparc*, a gene known to be critical in maintaining the integrity of skeletal and other connective tissue with age.^(^ ^88^, ^89^ ^)^

## Discussion

Our understanding of the genetic regulation of bone mass has been profoundly expanded through GWAS studies on BMD. The statistical power from large cohorts of individuals has identified candidate genes likely to regulate bone density and whose variance is therefore potentially involved in the development of osteoporosis.^(^ ^90^ ^)^ As important as these studies are, however, there is a need to functionally validate and characterize additional candidate genes, as there are a number of unmet needs in the management of osteoporosis, particularly in anabolic therapies.^(^ ^91^, ^92^ ^)^ Once the mechanistic role of each candidate gene in bone metabolism is established, then translational studies can aim to leverage these genetic determinants into therapeutic targets to alleviate bone loss, as has been done with romosozumab (*SOST*), denosumab (*RANKL*), and DKK1 inhibitors.^(^ ^93^, ^94^, ^95^ ^)^ As osteoporosis is a disease predominantly affecting the elderly, the need to understand how these candidate genes behave with age is equally important. However, many technical challenges impede the pipeline of functional validation from GWAS candidate genes, hindering our ability to understand how each gene mechanistically influences bone metabolism.

In this study, our aim was to simplify the progression from GWAS‐associated genes to the laboratory setting in studying age‐related bone loss. Studies with a similar aim have been performed by others, including the Alliston laboratory^(^ ^12^ ^)^; however, we sought to build upon previous work through the lens of single‐cell profiling. We first compared candidate gene expression in bone tissues across chronologically aged mice to determine which genes may have age‐associated functions in regulating bone mass. We found that many GWAS VNN genes exhibited upregulation in their expression with age, rather than downregulation. This could be due to a number of factors, including, but not limited to, increased bone turnover, upregulation of aging processes such as senescence, or increased transcription due to extrinsic factors influencing bone mass. Nevertheless, these age‐associated changes suggest that the affected genes have some role in the maintenance, or degradation, of bone tissue in aged mice. Interestingly, more candidate genes were upregulated in the bone tissue compared to the bone marrow. This indicates that age‐related gene expression changes may be occurring in more mature bone‐resident cells, such as osteocytes or osteoblasts, compared to stem or immune cells, which reside in the marrow. Of the genes found to be upregulated with age in both bone and marrow, nearly all of them have established roles in bone formation, such as *Sp7*,^(^ ^96^, ^97^ ^)^ *Tmem119*,^(^ ^98^ ^)^ *Bmp4*,^(^ ^99^ ^)^ and *Col1α2* ^(^ ^100^ ^)^; moreover, *Sp7* and *Col1α2* have established roles in vivo that indicate they are essential for musculoskeletal development. *Tmem119*, however, has only been characterized in vitro, while its role in organismal bone metabolism remains unclear. Although *Bmp4* is a member of the bone morphogenic protein family, which act as ligands stimulating bone formation, loss of *Bmp4* does not influence developmental skeletogenesis or fracture repair in adult mice.^(^ ^101^, ^102^ ^)^ However, *Bmp4* expression has been observed to increase with age^(^ ^103^ ^)^ and stimulate osteoclastic bone resorption,^(^ ^104^ ^)^ particularly when osteoblast‐derived.^(^ ^105^ ^)^ Additionally, polymorphisms in *Bmp4* have been associated with altered BMD in elderly individuals, at 70 to 85 years of age, with no association with fracture.^(^ ^106^ ^)^ This suggests that *Bmp4* may have a role in bone loss in aged mice and, perhaps, humans that remains incompletely understood.

It is important to note that the ages we used represent skeletally mature (6‐ to 9‐month‐old) and aged (21‐ to 27‐month‐old) mice, as this removes confounding variables from developmental changes observed before 4 to 6 months. Many candidate genes arising from GWAS studies are being validated in the International Mouse Phenotyping Consortium (IMPC) as well as independent laboratory studies. However, nearly all candidate gene knockout mice are being phenotyped at 4 to 6 months, which corresponds to middle age in humans and is typically before the onset of age‐related bone loss in both species.^(^ ^107^, ^108^, ^109^ ^)^ We wish to emphasize that this approach by the IMPC should not be mistaken as uninformed, as this age is typically when peak bone mass is observed in mice, and GWAS for BMD typically control for age as a covariate. Even so, due to the lack of an aging component in these follow‐up studies, the effects of many candidate genes on age‐related bone loss remain unknown.

At the single‐cell level, we found that GWAS VNN candidate gene expression was enriched in committed mesenchymal cells. Hypertrophic and prehypertrophic chondrocytes, osteo‐lineage cells, and mineralizing osteocytes harbored a majority of candidate gene expression, although other clusters such as Lepr^+^ MSCs, fibroblasts, and pericytes were enriched as well. Additionally, immune clusters in this dataset showed no enrichment of candidate gene expression, suggesting that these GWAS VNN genes likely act intrinsically within the mesenchymal lineage. These data provide a map for genes to be tested using cell‐specific mouse models to knock out each gene according to its cluster‐specific expression, as outlined in Table 1. This will be critical in designing functional validation experiments to study the mechanistic action of each candidate gene.

It is postulated that the main cause of impaired bone formation is either through (1) inhibited mesenchymal stem cell differentiation and commitment, leading to a reduced number of osteoblasts, or (2) intrinsically impaired osteoblast function, which reduces the osteoblast's capability of producing, depositing, and mineralizing extracellular bone matrix. For reasons given in what follows, our data suggest that a majority of candidate genes are likely to be more responsible for the latter function in regulating ECM proteins. In our GWAS VNN‐gene‐enriched cells, we found that the highest‐expressed non‐VNN gene was *Sparc*, which encodes the ECM protein osteonectin that is crucial for bone mass maintenance.^(^ ^110^ ^)^ Additionally, the highest expressed VNN genes across many clusters were *Mgp* and *Col1α2*, which encode ECM proteins. *Col1α2* is well known to be critically important for bone formation, and its mutation can lead to osteogenesis imperfecta.^(^ ^111^ ^)^ *Mgp*, however, remains an intriguing candidate gene for future study in bone. *Mgp*, or matrix gla protein, is closely related to osteocalcin (*Bglap: “bone gla protein”*), has been associated with chondrogenesis^(^ ^112^ ^)^ and arthritis,^(^ ^113^ ^)^ and promotes in vitro bone formation through Wnt signaling.^(^ ^25^ ^)^ In agreement with our data, expression of *Mgp* has been found in both chondrocytes and vascular cells, with its function differing in each cell type.^(^ ^114^ ^)^ Global loss of *Mgp* leads to premature death by 2 months of age through arterial calcification, so their true bone phenotype remains unknown. Therefore, cell‐specific targeting of *Mgp* in either chondrocytes or osteocytes may provide important data with regard to the role of *Mgp* in the maintenance of bone ECM and bone formation. Moreover, this co‐expression approach may be valuable in identifying noncoding RNAs that contribute to the regulation of BMD, as we found *Malat1—*a long noncoding RNA (lncRNA) that regulates osteogenesis^(^ ^37^, ^79^, ^80^, ^81^, ^115^ ^)^—as highly co‐expressed with VNN genes.

Illustrating the key regulatory subunits in the GWAS VNN‐gene‐enriched population, we identified *Sox9* as a crucial regulatory transcription factor within the osteo‐lineage cell population. The importance of *Sox9* has been demonstrated in neurogenesis^(^ ^116^ ^)^ and auditory hair cell development.^(^ ^117^ ^)^ *Sox9* is known to be a major fate determinant in MSCs that undergo chondrogenesis and osteoblastogenesis.^(^ ^118^ ^)^ We verified its importance for the osteoblast‐secreted SPARC protein, which enhances bone formation.^(^ ^110^ ^)^ In addition, mutations or polymorphisms in *Sparc* lead to idiopathic osteoporosis and osteogenesis imperfecta.^(^ ^110^, ^119^ ^)^

This study has several limitations. One is that the candidate genes used in our in silico analyses may not necessarily be causal to the associated genomic locus. GWAS‐associated loci typically lie in intergenic regions, making it difficult to determine which candidate gene underlies the association with BMD. Additionally, it has been shown that more than one gene may contribute to one genomic locus,^(^ ^120^ ^)^ further confounding candidate gene selection. Therefore, although our study thoroughly investigated over 400 associated genes, there may be other candidate genes associated with BMD that were not covered. However, even in such studies applying eQTL colocalization^(^ ^121^ ^)^ and chromatin conformation capture,^(^ ^122^ ^)^ this issue remains a factor, as the contributions of other genes cannot be excluded. For example, based on functional validation in cells and in vivo, the causal gene underlying the *CPED1‐WNT16‐FAM3C* locus (location: 7q31.31) has been assigned to both neighboring genes (*WNT16*,^(^ ^123^ ^)^ *FAM3C*,^(^ ^124^ ^)^ *CPED1* ^(^ ^125^ ^)^) and a gene identified through Capture C and ATAC‐Seq (*ING3* ^(^ ^122^ ^)^). This suggests that nearest‐neighbor genes probably cannot be excluded as contributing genes until proper functional validation. This is reflected in GWAS manuscripts, which list the nearest gene to each locus as a standard output. Moreover, in a recent study combining a transcriptome‐wide association study (TWAS) and eQTL localization to investigate causal GWAS genes, the authors found that this combinatory technique did not perform as well as prioritizing genes based on their proximity to GWAS loci.^(^ ^121^ ^)^ Thus, although we agree this is a limitation of our study, it is a limitation shared by the GWAS field and must not impede the downstream testing of candidate genes, which this manuscript seeks to facilitate. Additionally, the GWAS dataset we utilized may not capture a diverse genetic makeup, as the UK Biobank cohort consists largely of individuals of white ethnicity (94.6%). Nonetheless, candidate genes will continue to be predicted in future GWAS on new cohorts to address these limitations, so we reasoned that studies on any existing cohort will have these limitations. In line with our goal, our work established a framework that can be reapplied in future GWAS to accelerate the validation pipeline of candidate genes. Another limitation is that the differences found in the Tabula Muris Senis are mostly due to differences in the cellular composition of organs rather than transcriptional differences, even if cell‐type normalization and scaling reduce that bias.

In summary, this study provides data in translating the large number of GWAS‐associated candidate genes to a laboratory perspective for the study of age‐related bone loss. We found that GWAS VNN candidate genes were more likely to be upregulated with age and that many genes with age‐associated differential expression had unexplored roles in age‐related bone loss. Through enrichment of GWAS candidate gene expression in scRNA‐seq data, we categorized the top expressed genes by cell type and annotated Cre‐loxp systems for immediate use in the functional characterization of each candidate gene. We found that a majority of clusters expressing these genes were committed chondro‐ or osteogenic cell types. Of these cell types, the defining characteristics appeared to be the expression of ECM proteins, specifically *Sparc*. Additionally, the highest expressed GWAS VNN genes in these enriched cells appeared to encode ECM proteins, indicating that they were involved in the deposition and mineralization of bone matrix. Overall, this work provides further insights into the characterization of GWAS candidate genes and may help to bridge the gap between genetic and translational studies of osteoporosis, with the goal of streamlined development of therapeutic targets for the treatment of age‐related bone loss.

## Conflict of Interest Statement

The authors declare no conflicts of interest. The funders had no role in the design of the study; in the collection, analyses, or interpretation of data; in the writing of the manuscript; or in the decision to publish the results.

## Author Contributions

**Madison L. Doolittle:** Conceptualization; data curation; formal analysis; funding acquisition; investigation; methodology; validation; writing – original draft; writing – review and editing. **Sundeep Khosla:** Conceptualization; funding acquisition; investigation; resources; software; supervision; validation; writing – original draft; writing – review and editing. **Dominik Saul:** Conceptualization; data curation; formal analysis; funding acquisition; investigation; methodology; project administration; resources; software; supervision; validation; visualization; writing – original draft; writing – review and editing.

### Peer Review

The peer review history for this article is available at https://www.webofscience.com/api/gateway/wos/peer-review/10.1002/jbm4.10795.

## Supporting information


**Figure S1.** GSEA analysis of old versus young bone. Applying the selected GWAS‐VNN genes as a gene set, a core enrichment in the old bone (*A*, NES = 1.80, *p* = 0.0) and old marrow (*B*, NES: 1.47, *p* = 0.0) can be ascertained. In bone, the number of genes that rise with aging is significantly higher compared to the number of all genes (*C*, hypergeometric *p* value: 0.0039), while the difference is insignificant in the bone marrow (*D*, hypergeometric *p* value: 0.2892).

**Figure S2.** Composition of top 25% GWAS VNN‐gene‐enriched cells. The cellular composition of the highest GWAS VNN‐gene‐enriched cells shows that these are mainly Lepr^+^ MSCs, followed by fibroblasts, endothelial cells (ECs), hypertrophic chondrocytes, and mineralizing osteocytes.

**Figure S3.** Enrichment for significantly upregulated genes with aging (*n* = 52, *A*) or top 26 genes as in Fig. 1*F* (*B*) and top five genes as in Fig. 1*G* (*C*).

**Figure S4.** Regulon activity of all five regulons in combination (*A*), as well as separated into Nfe2 (*B*), Pparg (*C*), Sox17 (*D*), Runx3 (*E*), and Sox9 (*F*).

**Figure S5.** (*A*) Top 25% VNN (*B*) and non‐VNN co‐expressed (*C*) genes, demonstrated clusterwise on the *x*‐axis. The expression per cell cluster is depicted. The percentage of positive cells for the respective gene is represented on the *y*‐axis.

**Table S1.** 436 GWAS‐VNN candidate genes.

**Table S2.** Sample and cluster characteristics of bone and marrow scRNA‐seq datasets.

## Data Availability

Data sharing not applicable.
