# Supplementary figures and images for: Single‐Cell Integration of BMD GWAS Results Prioritize Candidate Genes Influencing Age‐Related Bone Loss
Source: JBMR Plus. 2023 Jul 7;7(10):e10795. doi: 10.1002/jbm4.10795 (PMC10556272; doi:10.1002/jbm4.10795)

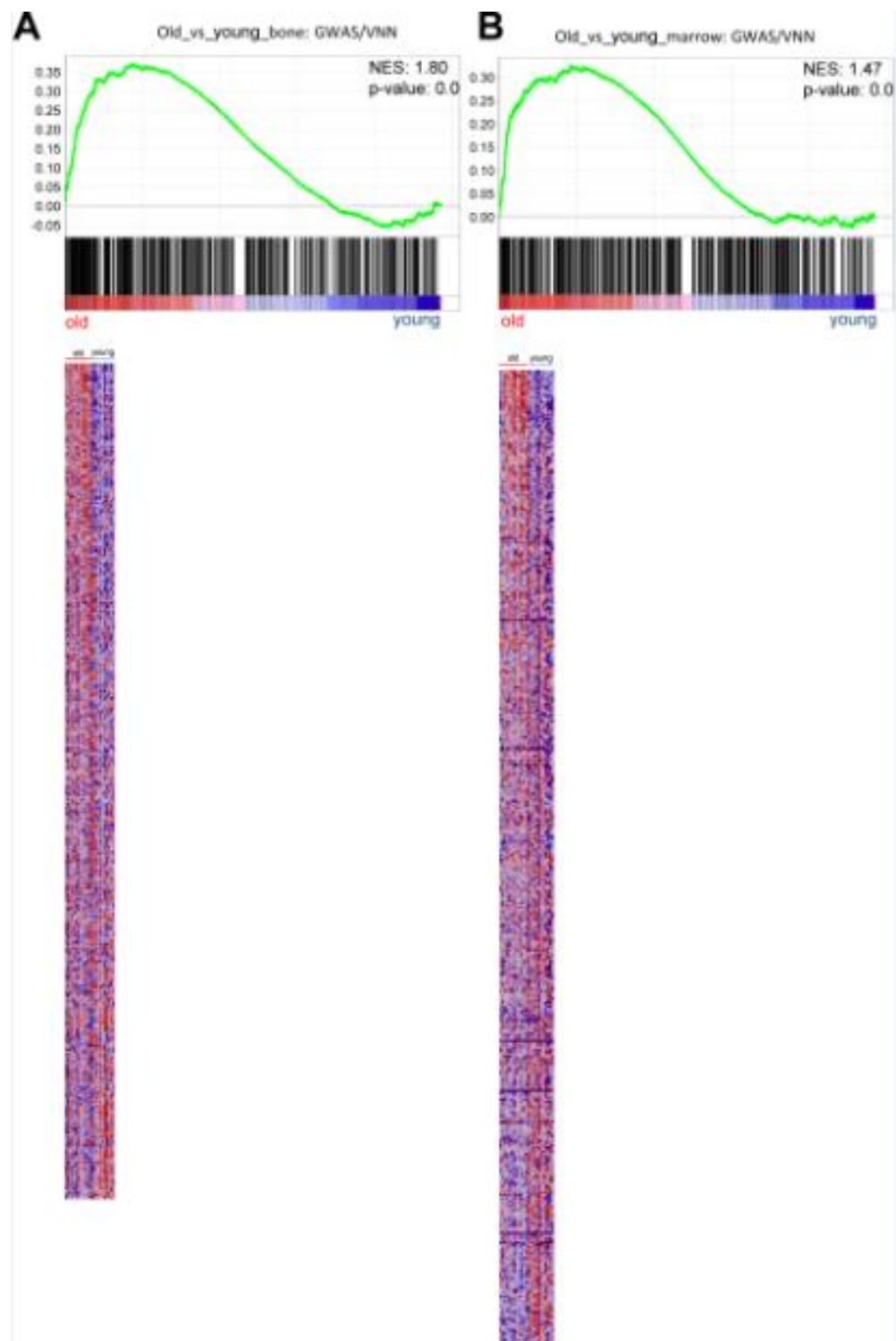

Supplement: Supplementary file 1 — Figure S1. GSEA analysis of old versus young bone. Applying the selected GWAS‐VNN genes as a gene set, a core enrichment in the old bone (A, NES = 1.80, p = 0.0) and old marrow (B, NES: 1.47, p = 0.0) can be ascertained. In bone, the number of genes that rise with aging is significantly higher compared to the number of all genes (C, hypergeometric p value: 0.0039), while the difference is insignificant in the bone marrow (D, hypergeometric p value: 0.2892). [file JBM4-7-e10795-s001.pdf]

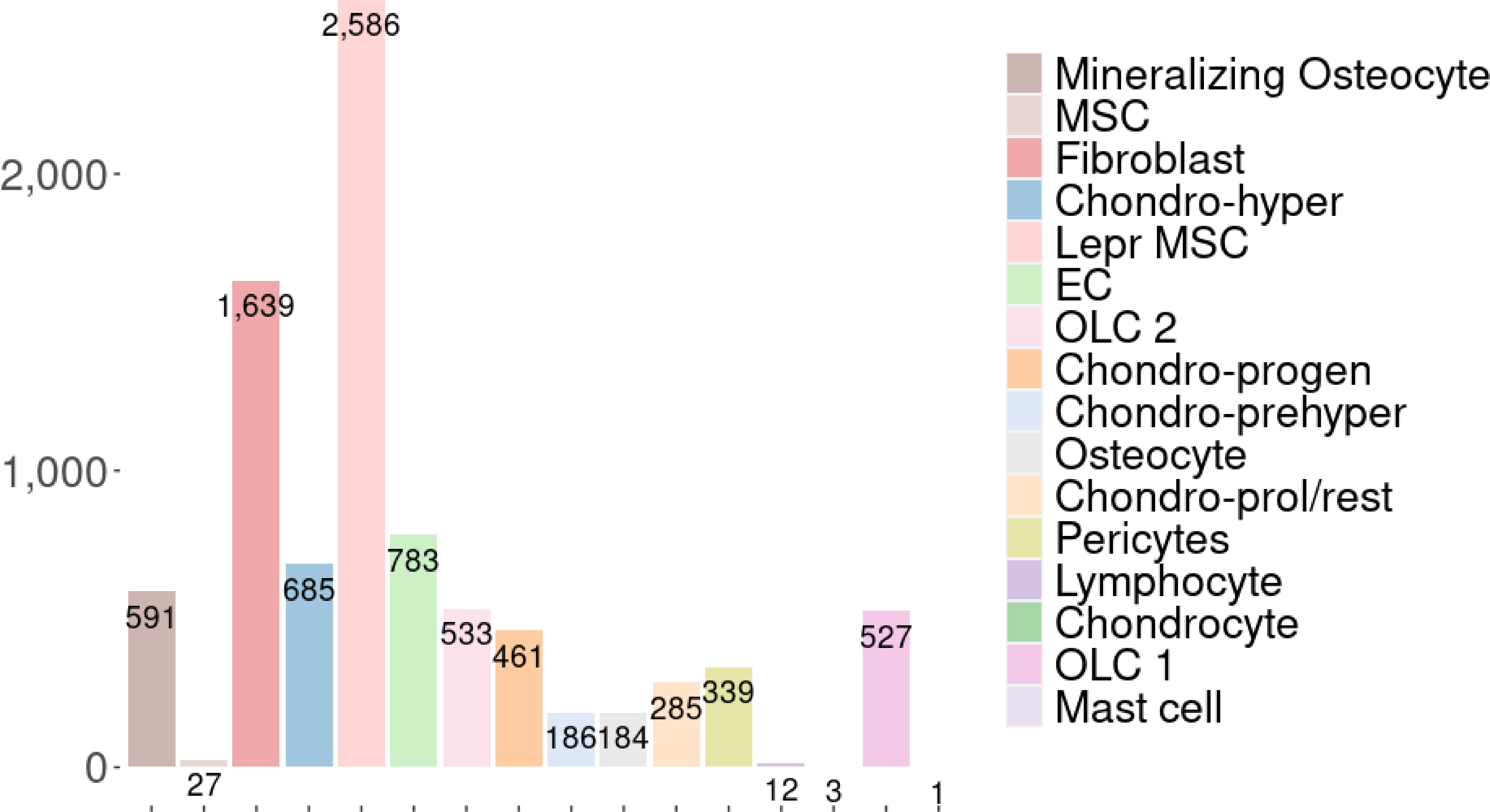

Supplement: Supplementary file 2 — Figure S2. Composition of top 25% GWAS VNN‐gene‐enriched cells. The cellular composition of the highest GWAS VNN‐gene‐enriched cells shows that these are mainly Lepr+ MSCs, followed by fibroblasts, endothelial cells (ECs), hypertrophic chondrocytes, and mineralizing osteocytes. [file JBM4-7-e10795-s006.tif]

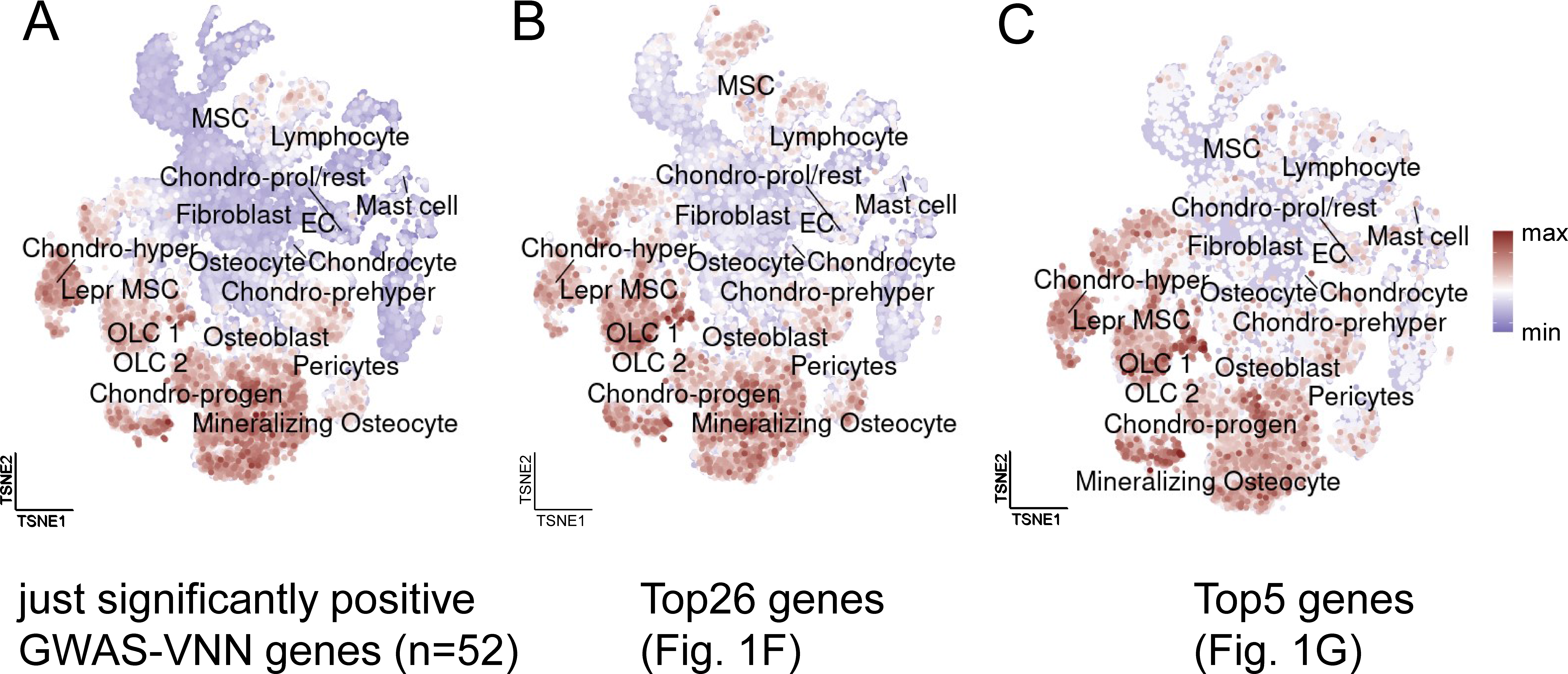

Supplement: Supplementary file 3 — Figure S3. Enrichment for significantly upregulated genes with aging (n = 52, A) or top 26 genes as in Fig. 1F (B) and top five genes as in Fig. 1G (C). [file JBM4-7-e10795-s004.tif]

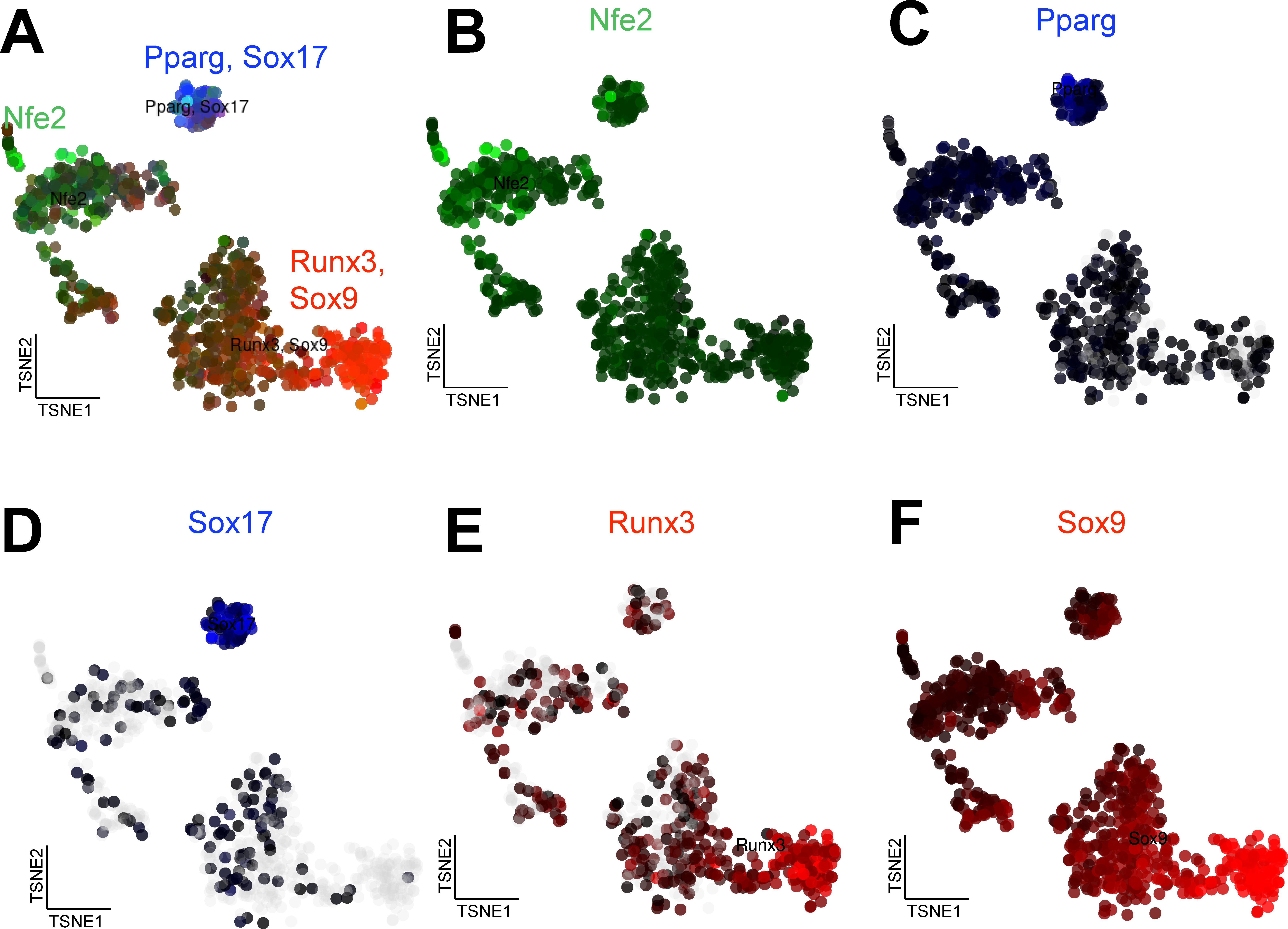

Supplement: Supplementary file 4 — Figure S4. Regulon activity of all five regulons in combination (A), as well as separated into Nfe2 (B), Pparg (C), Sox17 (D), Runx3 (E), and Sox9 (F). [file JBM4-7-e10795-s007.tif]

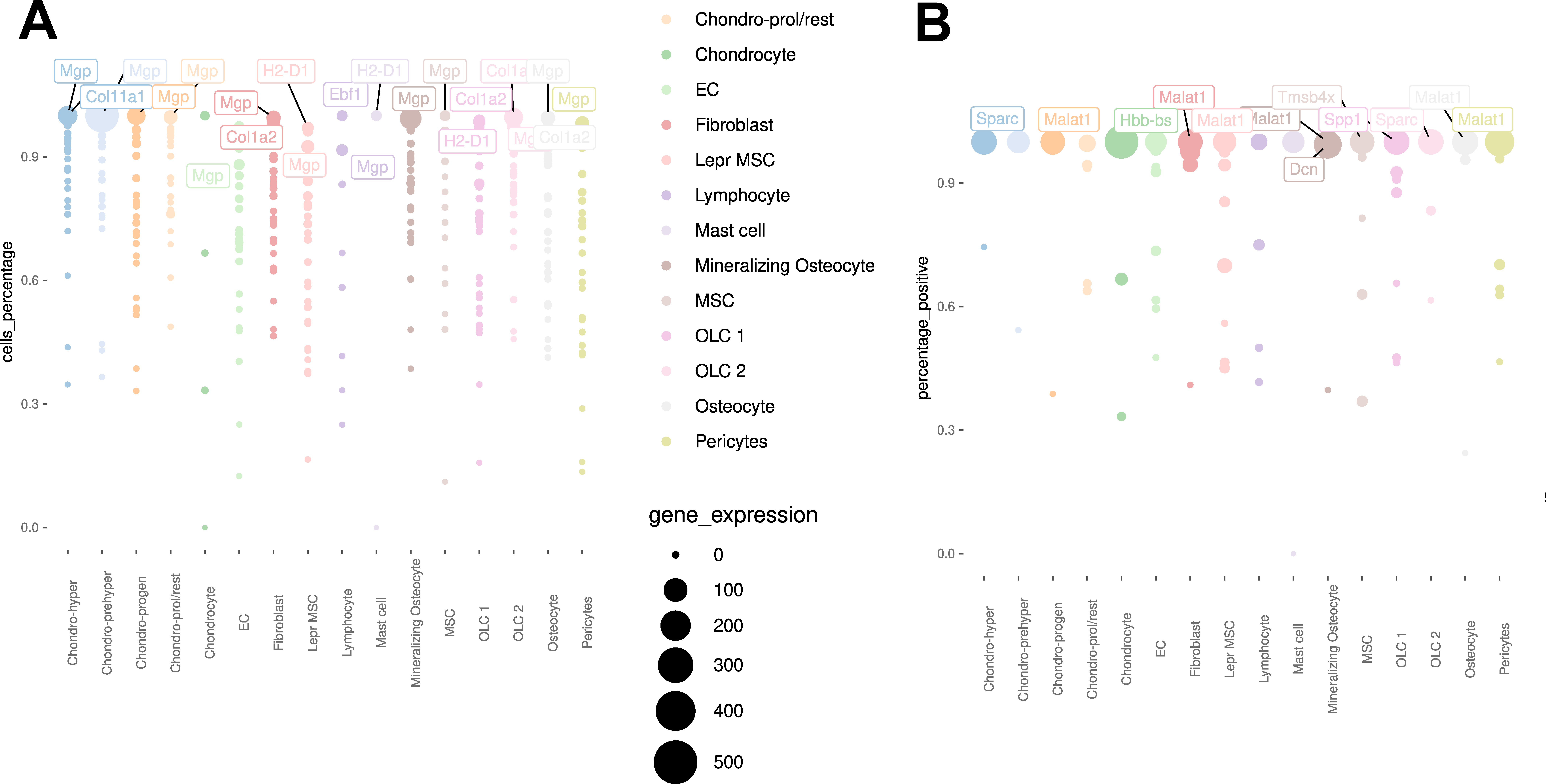

Supplement: Supplementary file 5 — Figure S5. (A) Top 25% VNN (B) and non‐VNN co‐expressed (C) genes, demonstrated clusterwise on the x‐axis. The expression per cell cluster is depicted. The percentage of positive cells for the respective gene is represented on the y‐axis. [file JBM4-7-e10795-s003.tif]
